# T cell heterogeneity in asthma pathogenesis: from immunological mechanisms to biological targeted therapies

**DOI:** 10.3389/fimmu.2025.1658774

**Published:** 2025-12-11

**Authors:** Qifeng Gan, Yuzhen Zhu, Yuxin Guo, Guo Fu, Xiao-Bin Zhang

**Affiliations:** 1 Department of Pulmonary and Critical Care Medicine, Zhongshan Hospital of Xiamen University, School of Medicine, Xiamen University, Xiamen, Fujian, China; 2 Department of Hematology, The First Affiliated Hospital and Institute of Hematology, School of Medicine, Xiamen University, Xiamen, Fujian, China

**Keywords:** asthma, CD4+ T cell, Th1/Th2, ILC2, Th17/Treg, targeted therapy

## Abstract

Severe and overlapping asthma endotypes—particularly steroid-insensitive disease—remain undertreated, highlighting the need for a T-cell–centered synthesis. This review frames asthma heterogeneity through the interplay of T-cell axes, with Th2 pathways shaping T2-high disease and Th17/Treg imbalance characterizing T2-low features and much of steroid resistance. Building on this framework, we map therapies to mechanism: IL-4Rα and IL-5/IL-5R blockade chiefly mitigate Th2-dominated circuits, whereas upstream alarmin inhibition (e.g., TSLP) modulates epithelial–immune cues that influence both T2-high biology and selected T2-low processes. We then outline what is needed to translate mechanisms into decisions—integrated biomarkers to refine endotypes and mechanism-guided switching or combinations, with emphasis on T2-low populations where unmet need is greatest. By linking T-cell biology to therapeutic leverage points, the review offers a concise path from mechanism to patient stratification and more rational treatment choices.

## 1 Introduction

Bronchial asthma (asthma) is a heterogeneous disease characterized by airway hyperresponsiveness (AHR), chronic airway inflammation, variable expiratory airflow limitation, and respiratory symptoms including cough, chest tightness, wheezing, and dyspnea. Its pathogenesis is closely associated with allergen exposure, air pollution, viral infections, sex hormones, and neuroregulation (1, 2). Asthma remains a prevalent global health concern, affecting 1–29% of populations worldwide (2, 3). Asthma is a heterogeneous disease in which clinical variability reflects distinct immunologic endotypes rather than a single pathway. Approximately half of asthma patients exhibit T2-high (T2-high) asthma, also known as eosinophilic asthma (5, 6). In T2-high disease, epithelial alarmins (TSLP/IL-33/IL-25) condition dendritic cells and other antigen-presenting cells to prime Th2 responses and license ILC2 (Group 2 Innate Lymphoid Cells) activation, driving eosinophilic inflammation, mucus hypersecretion and corticosteroid-responsive pathology (4–7). By contrast, T2-low disease features Th17/Treg disequilibrium with neutrophilic inflammation, steroid insensitivity, and contributions from environmental and metabolic cues that sustain non-type-2 pathways (6–8). Among diverse immune mediators, T lymphocytes integrate epithelial alarmins and antigen-presenting-cell signals to shape both allergic (T2-high) and non-allergic (T2-low) endotypes. This motivates a focused review on T-cell–centered mechanisms and their translational leverage points across current and emerging therapies.

## 2 Mechanisms of T lymphocyte-mediated asthma pathogenesis

### 2.1 Th1/Th2 imbalance

Th2 dominance is a hallmark of allergic asthma, driven by epithelial and antigen-presenting cells (APCs) cues that favor IL-4/IL-13 programs under GATA3/STAT6 (GATA Binding Protein 3/Signal Transducer and Activator of Transcription 6) control, whereas insufficient or ineffective Th1 activity can coexist with severe disease and contribute to steroid-insensitive features in selected contexts (9–11). Thus, endotypes are best understood as a dynamic balance between Th2 effector circuits and counter-regulatory Th1 signals rather than isolated molecules (10–12). To make this balance operational, it helps to follow the initiating sequence from epithelium to T-cell fate. In the paragraphs below, we trace how upstream cues are translated into Th2 programs and where reciprocal Th1 signals constrain this trajectory (9). Alarmins (TSLP/IL-33/IL-25) instruct dendritic cells and other APCs to prime Th2 cells and amplify IgE class-switch via Tfh, while ILC2 rapidly reinforces type-2 cytokine output; downstream, IL-4/IL-13 promote eosinophil recruitment, goblet-cell hyperplasia and AHR (9–12) (**Figure 2**). Reciprocal IFN-γ/STAT1 signaling constrains GATA3 programs and limits mucus metaplasia, but pollutant and infection cues can tip the axis back toward Th2 (13–15). Of note, PM2. 5 (particulate matter ≤2. 5 µm aerodynamic diameter) and viral triggers heighten epithelial cytokines and co-stimulation, reshaping APC polarization and re-skewing Th responses (13–16). Approved biologics already intersect this circuitry: IL-4Rα blockade dampens Th2/Tfh pathways and IgE biology, while IL-5/IL-5R targeting reduces eosinophil-linked effector loops that sustain Th2 inflammation (9–12). At the level of axis re-balancing, strategies to modulate GATA3 or augment IL-12/IFN-γ have been explored as means to reinforce Th1 counter-regulation (with endotype-guided selection and safety monitoring) (13, 14). Upstream alarmin inhibition (e. g., TSLP) may complement these approaches by resetting epithelial–APC cues that initiate Th2 polarization (15, 16). Beyond the classical dichotomy, context-dependent plasticity introduces overlap between Th2 and Th17 programs—including hybrid Th17/Th2 features observed in severe or steroid-insensitive disease; detailed mechanisms and clinical implications are discussed in Section 2. 3 (Th17/Treg) (9–16).

### 2.2 ILC2s synergistically regulate immune responses in T2-high asthma

While Th2 cells orchestrate antigen-dependent adaptive responses, ILC2s are poised to mount a rapid, antigen-independent type-2 program in response to epithelial stress, thereby shaping the context in which Th2 immunity unfolds (17–20). ILC2s lack a T-cell receptor and are activated directly by epithelial alarmins (TSLP/IL-33/IL-25), neuro-immune cues and tissue signals, enabling an early, tissue-resident burst of type-2 cytokines independent of antigen priming (18–21, 24, 25). Their cytokine output overlaps with Th2 (IL-5, IL-13) but is distinct in important ways: ILC2s are a prominent source of IL-9, supporting goblet-cell metaplasia and mast-cell fitness, and they rapidly amplify local inflammation before antigen-experienced Th2 cells arrive (19–21) (**Figure 2**). In addition, ILC2s can express MHC-II, allowing peptide presentation to CD4^+^ T cells, and constitutively express PD-L1; engagement of PD-L1–PD-1 on CD4^+^ T cells promotes GATA-3 up-regulation and Th2 polarization, highlighting an antigen-independent route by which ILC2s program adaptive immunity (22, 23). Upon allergen exposure, alarmin-conditioned APCs (APC, antigen-presenting cell) prime Th2/Tfh responses in lymph nodes, whereas ILC2s respond *in situ* within minutes to hours, releasing IL-5/IL-13 (and IL-9) that recruit eosinophils, enhance DC migration, and prepare the tissue niche for incoming Th2 cells (17, 20, 21). This temporal and mechanistic complementarity—ILC2s as initiators/amplifiers and Th2 as orchestrators of antigen-specific expansion—explains the robustness of type-2 inflammation and its clinical correlates (mucus hypersecretion, AHR, eosinophilia) (17, 20, 21). Neuropeptidergic inputs such as CGRP(calcitonin gene-related peptide) further tune ILC2 activity and can be leveraged experimentally to dampen ILC2-dependent pathology (24, 25). Importantly, T2-high asthma is not monolithic: early-onset atopic disease with strong IgE/Tfh features can differ from late-onset eosinophilic phenotypes in the degree of ILC2 vs Th2 dominance and upstream epithelial drivers (17, 26). Recognizing these overlapping yet distinct mechanisms explains variable responses to biologics that target IL-4Rα/IL-13, IL-5/IL-5R, or upstream TSLP, and motivates biomarker-guided selection and switching (26).

### 2.3 Th17/Treg imbalance

In contrast to eosinophilic, Th2-dominant disease, T2-low/neutrophilic asthma is closely linked to a higher Th17/Treg ratio, airway obstruction, and corticosteroid-insensitive features (27–29). This balance provides a mechanistic lens for endotyping beyond simple Th1/Th2 dichotomy (27–29).

**Figure 1:**
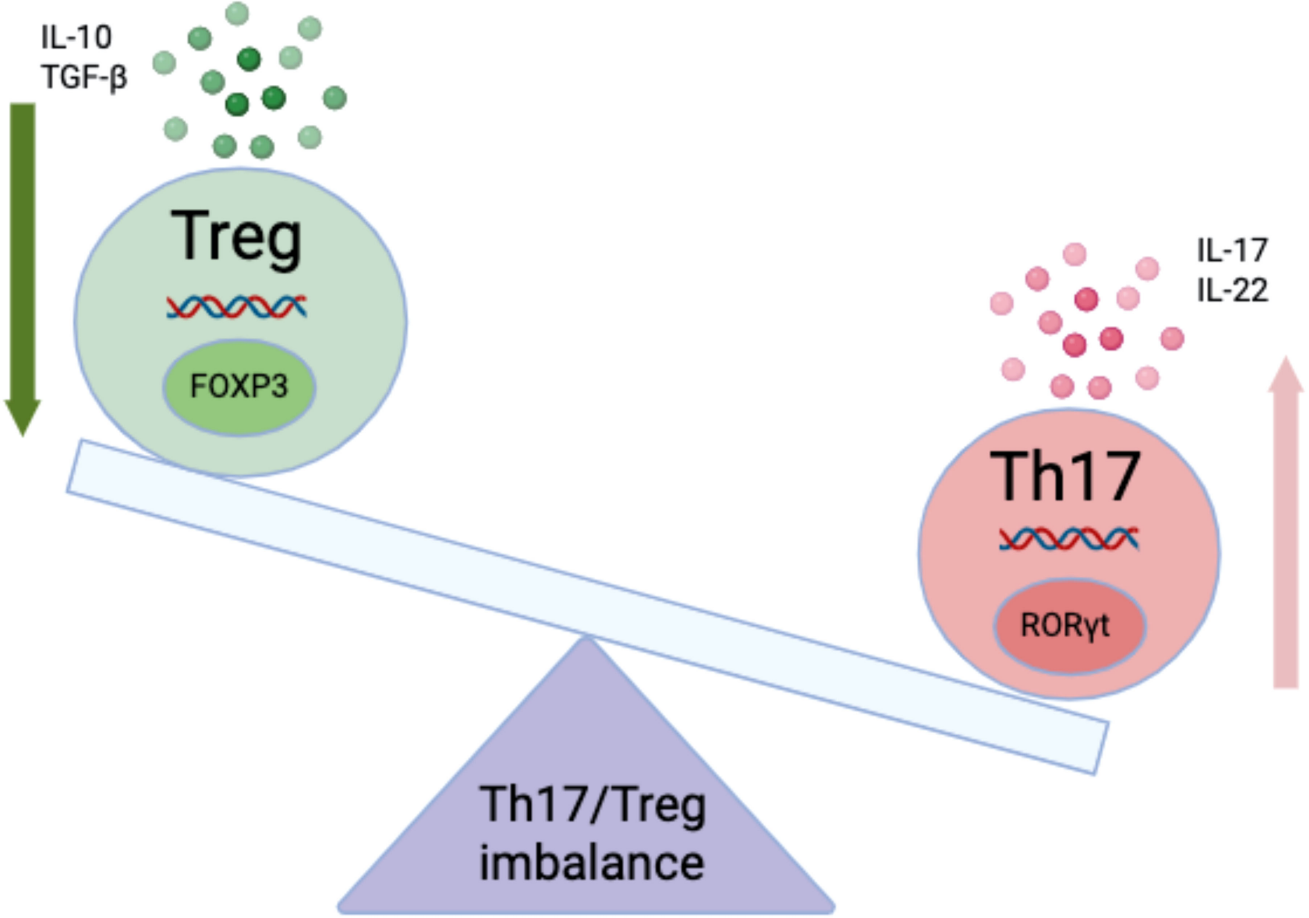
Coordinated regulation of type-2 immune responses by Th2 cells and ILC2s in T2-high asthma. Schematic of how epithelial injury/stress (allergens, air pollution, viral infection) releases TSLP, IL-33, and IL-25, which condition dendritic cells to prime naïve CD4^+^ T cells into Th2 via OX40L–OX40 and directly activate ILC2s. Activated ILC2s provide a rapid, antigen-independent burst of IL-5/IL-13 (and IL-9) that recruits eosinophils, enhances DC trafficking, and prepares the tissue niche before antigen-experienced Th2 cells expand. Th2 (with Tfh help) promotes IgE class-switching in B cells and drives mucus hypersecretion, goblet-cell hyperplasia, and airway hyperresponsiveness, while mast-cell mediators (e.g., PGD_2_, LTD_4_) and eosinophil-derived signals sustain type-2 inflammation and contribute to airway remodeling. The figure emphasizes temporal complementarity—ILC2s as rapid initiators/amplifiers and Th2 as antigen-specific organizers—and the upstream epithelial–immune axis that underlies variable responses to IL-4Rα/IL-5/IL-5R and TSLP-targeted therapies. DC, dendritic cell; Tfh, T follicular helper cell; ILC2, group-2 innate lymphoid cell; PGD_2_, prostaglandin D_2_; LTD_4_, leukotriene D_4_.

**Figure 2:**
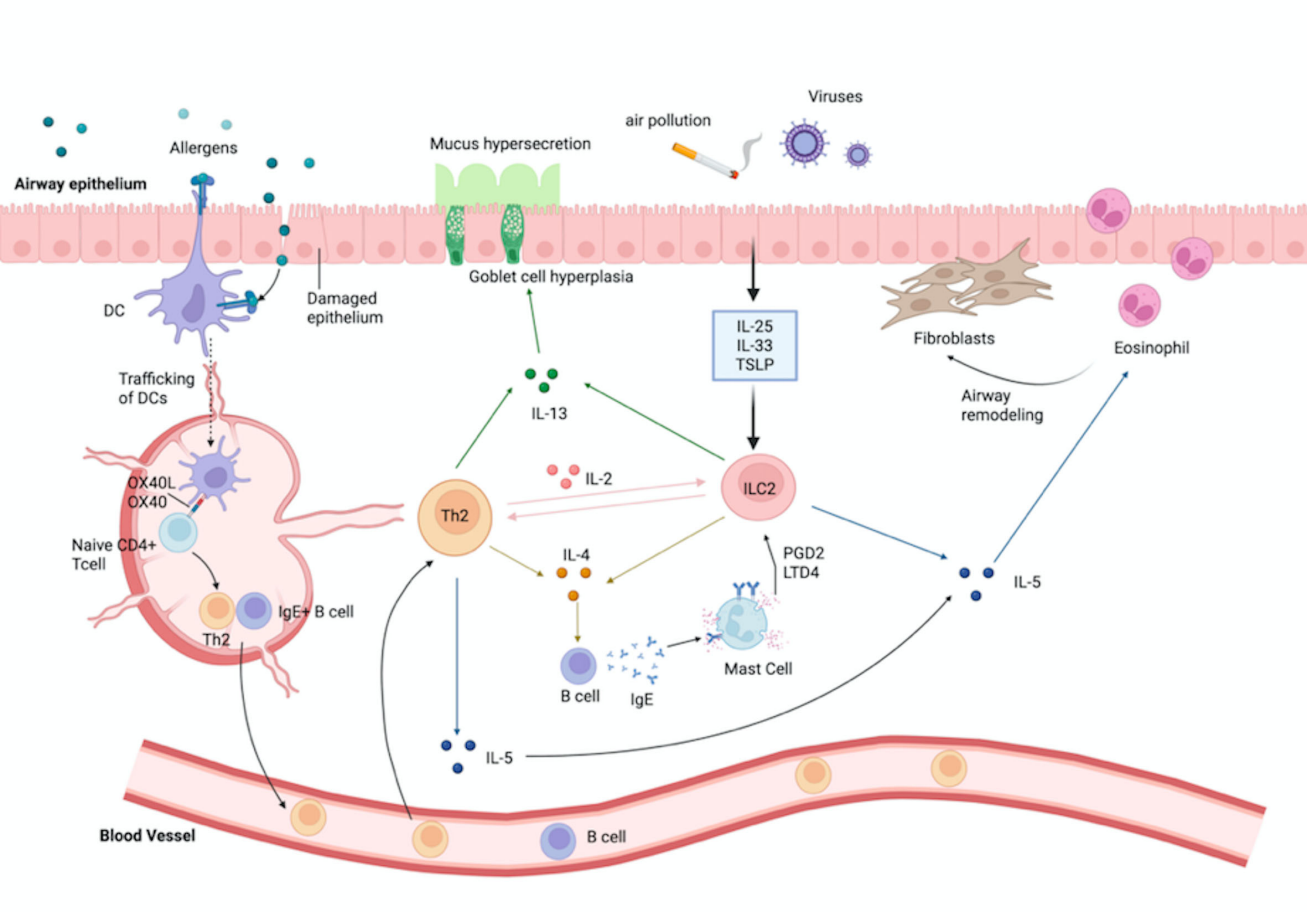
Th17/Treg imbalance in asthma pathogenesis. Conceptual balance between FOXP3^+^ regulatory T cells (Treg)—which release IL-10 and TGF-β to restrain airway inflammation—and RORγt^+^ Th17 cells, which produce IL-17 and IL-22 and are linked to neutrophilic inflammation and corticosteroid insensitivity. The seesaw illustrates how a shift toward Th17 dominance (upward arrow) with relative Treg contraction (downward arrow) aligns with T2-low features and airflow limitation, providing a mechanistic lens for endotyping beyond the classical Th1/Th2 dichotomy. FOXP3, forkhead box P3; RORγt, RAR-related orphan receptor gamma t.

Beyond antagonism, Th lineages display context-dependent plasticity: Th17 cues (e.g., IL-6/IL-1β/IL-23) can imprint IL-17–producing Th2 or hybrid Th17/Th2 states, whereas type-2 milieus can reciprocally modulate Th17 programs (27–30). These overlaps help explain mixed granulocytic phenotypes and variable steroid responsiveness (27–30). Mechanistically, the emergence of IL-17^+^ Th2 and Th17↔Th2 hybrid states is not stochastic; it is fostered by upstream contexts that promote Th17 programming while eroding Treg stability. To make these influences explicit, we next group the determinants into environmental, metabolic, and cytokine drivers that create a milieu permissive for plasticity and disease persistence (31–33). Environmental: particulate pollution and respiratory pathogens enhance epithelial alarmins and dendritic-cell signals that favor IL-17 responses and reduce Treg stability (31, 32). Metabolic: obesity-associated mediators (e.g., leptin) and mitochondrial stress shift differentiation toward Th17 while constraining FOXP3^+^ Treg function (**Figure 2**) (33, 34). Cytokine network: IL-6^+^ TGF-β initiate Th17, while IL-23 maintains pathogenic programs that are relatively glucocorticoid-refractory (27, 32). A Th17-skewed/Treg-deficient axis aligns with neutrophilic inflammation (**Figure 1**), steroid resistance, and suboptimal responses to Th2-targeted biologics; biomarker-guided strategies are therefore needed to identify patients for alternative or combination approaches (27, 33). Although evidence is preclinical, Ephedrae Herba polysaccharides (EHP, botanical polysaccharide fractions isolated from Ephedra (mahuang)), lower IL-17A, increase TGF-β/IL-10, expand Treg, and attenuate airway inflammation in OVA models—changes that mechanistically align with a treat-to-endotype rationale for T2-low/steroid-insensitive disease (31–33). If translated, Th17/Treg-modulating agents such as EHP could serve as adjuncts to standard therapy in biomarker-enriched populations (e.g., high IL-17/Treg ratio); however, dose standardization, pharmacokinetics, herb–drug interaction screening, and safety evaluation remain prerequisites for clinical testing (31–33). ,

### 2.4 Tfh cells activity is mechanistically linked to asthma onset

Beyond Th1/Th2, Th2/ILC2 and Th17/Treg axes, humoral immunity critically shapes allergic asthma: Tfh cells license germinal-center reactions and IgE class switching, whereas Bregs counter-regulate this process via IL-10/TGF-β and contact-dependent mechanisms. Thus, the Tfh–Breg balance is a fourth pillar connecting T-cell biology to antibody-mediated pathology (34–36, 38–40). Tfh differentiation and function require Bcl-6 and IL-21, with IL-4 from Tfh promoting IgE class switching; ICOS–ICOSL and PD-1–PD-L1 interactions stabilize Tfh–B-cell synapses and sustain plasma-cell outputs (34–37, 40). Bregs (IL-10/TGF-β-producing) curb this circuit by limiting excessive Tfh activation and restraining IgE overproduction, thereby maintaining humoral homeostasis (38, 39). When Tfh activity increases and Breg function contracts, the net effect is heightened IgE production and amplification of allergic inflammation (38–40). The magnitude of the Tfh–Breg imbalance tracks clinical heterogeneity: early-onset atopic/T2-high asthma typically shows a stronger Tfh–IgE axis, whereas phenotypes with overlapping upper-airway disease may display persistent IgE outputs despite standard therapy, underscoring a need for strategies that rebalance Tfh/Breg rather than only suppress type-2 cytokines (36, 38–40). Experimental and clinical observations indicate that reducing Tfh activity and/or restoring Breg function parallels improvements in symptoms and biomarkers (38, 39). Taken together, these phenotype-linked patterns indicate that therapies which dampen Tfh output and/or restore Breg function are most likely to benefit atopic/T2-high endotypes in whom IgE/Tfh signatures persist despite standard care (36, 38–40). In this context, recombinant Alt a 1 allergen immunotherapy represents a mechanism-anchored option to recalibrate the Tfh/Breg axis (36, 38–40). Recombinant Alt a 1 allergen immunotherapy not only decreases allergen-specific IgE but also recalibrates the Tfh/Breg axis—down-modulating Tfh activation and enhancing Breg-mediated IL-10/TGF-β signals—thereby attenuating airway inflammation (39). These effects provide a mechanism-anchored rationale for AIT as an adjunct in atopic/T2-high phenotypes characterized by a dominant Tfh–IgE program, with the potential to complement biologics targeting IL-4/IL-13 when IgE/Tfh signatures remain high (36, 38–40). Future work should prospectively stratify patients using Tfh/Breg-linked biomarkers to test whether Tfh/Breg re-balancing predicts clinical benefit (38–40).

### 2.5 Cytotoxic T lymphocytes

CD8^+^ T cells contribute to airway pathology through functionally distinct subsets. Tc2 cells (IL-5/IL-13) potentiate eosinophilia, mucus hypersecretion, and airway hyperreactivity, mirroring type-2 circuits (41). Tc9 cells (IL-9) support mast-cell survival and epithelial remodeling, thereby amplifying mucus responses and barrier changes (41, 42). Tc17 cells (IL-17A/F) promote neutrophilic inflammation and are frequently linked to glucocorticoid insensitivity (42, 43). In contrast, Tc22 cells (IL-22) primarily act on the epithelial barrier with context-dependent effects on repair versus inflammation (43). A regulatory arm—CD8^+^ Tregs (IL-10/TGF-β)—can suppress pathogenic T-cell and B-cell activity and restrain airway inflammation (41–43). Together, these profiles clarify how CD8^+^ T cells can either drive inflammation (Tc2/Tc9/Tc17) or promote restraint/repair (Tc22/CD8^+^ Tregs) in asthma (41–43). Recent data indicate that S100A4 marks a dysfunctional effector-memory CD8^+^ T cells state in asthma in which mitochondrial metabolism is impaired, leading to reduced T-bet/IFN-γ output and defective cytotoxic function (41, 43). This bioenergetic shift weakens CD8-mediated antiviral/immune-regulatory control and may exacerbate airway inflammation when Tc2/Tc17 programs dominate (41, 43). Conversely, experimental restoration of mitochondrial fitness (e. g., enhancing oxidative phosphorylation) can partially rescue IFN-γ competence, supporting a mechanistic link between metabolic stress and loss of CD8^+^ T cells restraint (43). Mapping CD8^+^ T cells states to mechanism suggests that Tc17 skewing plus S100A4-associated IFN-γ deficiency may cooperate to sustain neutrophilic, steroid-refractory disease, whereas Tc2/Tc9 activity amplifies type-2 pathology (42, 43). These insights argue for endotype-informed strategies that pair upstream biologics with metabolic rescue of CD8^+^ T cells or approaches that limit Tc2/Tc17 outputs, especially in patients with persistent exacerbations despite guideline therapy (41–43).

## 3 Clinical translation based on asthma immunological mechanisms

### 3.1 Comparison among well-established targeted therapeutic agents

As the immunopathogenesis of asthma has been clarified, targeted therapies have progressed rapidly, showing clinical benefit by modulating defined inflammatory pathways (44–84). **Table 1** synthesizes these data. Several conclusions emerge with direct implications for practice:(1)Greatest clinical impact—axis-matched biologics. Agents directed at IgE (omalizumab), IL-5/IL-5R (mepolizumab/reslizumab/benralizumab), and IL-4Rα (dupilumab) consistently reduce exacerbations and oral-corticosteroid (OCS) exposure in T2-high/eosinophilic or allergic endotypes when used with appropriate biomarker selection (baseline IgE/sensitization, blood eosinophils, FeNO) (47, 50–59);(2)Upstream breadth with TSLP. Tezepelumab acts at the epithelial–APC interface and improves outcomes across eosinophil strata, including some T2-low patients—making it a reasonable option when T2 signals are low or discordant (48, 54, 62–64); (3) Biomarker-guided choice and switching are essential. Aligning therapy with the dominant axis (IgE, eosinophils/IL-5, IL-4/IL-13, alarmins) and reassessing after an adequate trial supports rational selection, switching, or combination strategies (47, 52, 54, 56–59); (4) Safety/route considerations matter. SC vs IV administration, anaphylaxis observation (anti-IgE), transient eosinophilia/ocular events (IL-4Rα), and helminth precautions (IL-5Rα) influence long-term use (50–55). These observations support selection, switching, or combination based on the dominant axis. Against this backdrop, IL-9–directed therapy highlights the distinction between validated and still-exploratory pathways: agents in **Table 1** deliver consistent benefit in biomarker-defined endotypes, whereas IL-9 sits at the margin of the Th2/Tc9/ILC2 network with less certain prevalence and weaker biomarker guidance (44, 66, 67). Regarding anti-IL-9, preclinical murine studies showed marked suppression of airway remodeling (↓TGF-β1 68%, ↓VEGF 55%) with improved peripheral airway function (44). These observations underscore the need for endotype-enriched enrollment and mechanistic biomarkers when testing agents beyond the well-validated axes. **Table 1** therefore organizes established therapies by targeted pathway, responsive endotype/biomarker, key clinical effects, and caveats, providing a practical map from mechanism to treatment decisions.

**Table 1:** More mature targeted drugs for asthma.

More mature targeted drugs for asthma						
Targeted drugs	Mechanism of action	Research progress	Real-world data	Target population	Adverse reactions	References
Anti-IgE monoclonal antibody (Omalizumab)	Neutralizes free IgE and down-regulates FcϵRI on mast cells/basophils and dendritic cells, thereby weakening Tfh-driven IgE class switching and downstream Th2 amplification (links to the Tfh–Th2 axis).	Approved for moderate-to-severe allergic/T2-high asthma; dosing guided by baseline IgE and body weight.	For patients with moderate-to-severe allergic asthma, omalizumab treatment significantly reduces the rate of asthma exacerbations, improves lung function and overall asthma control, and enhances quality of life. Real-world evidence supports this efficacy; for instance, one study reported an approximately 50% reduction in the annual asthma exacerbation rate among treated patients.	Patients with moderate-to-severe allergic asthma who have elevated IgE levels (≥30 IU/mL) and positive skin prick test results.	The most common adverse effects are injection site reactions. Serious hypersensitivity reactions are rare, occurring in fewer than 0. 1% of patients.	(45–49)
Anti-IL-5/IL-5Rα monoclonal antibodies (Reslizumab, Benralizumab)	Anti-IL-5Rα:Blocks IL-5, reducing eosinophil maturation/survival and breaking the Th2/Tc2–eosinophil effector loop; indirectly lowers type-2 cytokine reinforcement; Anti-IL-5:Neutralizes IL-5 to reduce eosinophil load and the downstream Th2/Tc2 effector axis that drives exacerbations.	Benralizumab and Reslizumab are approved for severe eosinophilic asthma; novel long-acting formulations (e. g., reslizumab) are under investigation.	Real-world studies demonstrate that anti-IL-5/IL-5R biologics reduce exacerbation rates by approximately 50% and decrease oral corticosteroid requirements, with the most pronounced effects in patients exhibiting elevated blood or sputum eosinophils. For instance, benralizumab shows rapid eosinophil depletion and symptom improvement in real-world settings, while some studies observed long-term stabilization of lung function (FEV_1_)	Severe eosinophilic asthma (blood eosinophils ≥300 cells/μL)	Headache, arthralgia; rarely eosinophilic granulomatosis with polyangiitis (EGPA)	(47, 50–55)
IL-4/IL-13 inhibitors (Dupilumab):	Inhibits IL-4/IL-13 signaling via IL-4Rα; directly dampens Th2 differentiation and Tfh-mediated IgE class switching, and reduces mucus/epithelial remodeling signals.	Currently approved for treating moderate-to-severe eosinophilic asthma and atopic dermatitis	In real-world settings, dupilumab demonstrates significant efficacy in patients with elevated eosinophils and fractional exhaled nitric oxide (FeNO), reducing exacerbation frequency and improving lung function. Some studies report additional benefits for patients with severe asthma comorbid with nasal polyps or atopic dermatitis;	Th2-high phenotype asthma (characterized by blood eosinophils ≥150 cells/μL and/or FeNO ≥25 ppb);	Conjunctivitis, injection site reactions, and eosinophilia.	(47, 52, 54, 56–59)
Anti-leukotriene agents (e. g., the leukotriene receptor antagonist zafirlukast).	They inhibit the binding of leukotrienes (such as LTB4, LTC4) to their receptors, thereby reducing bronchoconstriction, mucus secretion, and inflammatory cell infiltration	These agents are widely used in clinical practice. Recent research has focused on optimizing combination therapy (e. g., with inhaled corticosteroids (ICS)) and personalized medication regimens.	Comprehensive real-world evidence documenting specific outcomes remains limited in the recent literature.	Patients with mild-to-moderate asthma, especially those with aspirin-exacerbated respiratory disease (AERD) and exercise-induced bronchoconstriction (EIB)	Headache and gastrointestinal disturbances; rare neuropsychiatric symptoms (such as depression, anxiety)	(60, 61)
TSLP Inhibitor (Tezepelumab)	Neutralizes TSLP to block epithelial→APC priming (e. g., DC OX40L) and limit ILC2 activation and Th2 polarization; provides upstream control that can extend into selected T2-low biology.	Approved by the FDA in 2021 for severe asthma regardless of phenotype, including low-eosinophil subgroups, it represents the first asthma-targeted biologic therapy directed against an epithelial cytokine.	Preliminary observations indicate Tezepelumab reduces exacerbations and improves symptoms, with potential advantages noted particularly in patients without significantly elevated blood eosinophil counts or fractional exhaled nitric oxide (FeNO). However, real-world evidence is currently limited, and further research is required to validate its efficacy across diverse biomarker-defined subgroups.	Severe asthma (irrespective of eosinophil levels).	Upper respiratory tract infections, injection site reactions.	(48, 54, 62–64)
*Anti-IL-33 antagonists(Itepekimab)	Inhibits IL-33 signaling, reducing ILC2 early amplification and subsequent Th2/Tfh polarization (alarmin→T-cell axis)	Investigational; Phase II clinical trial phases; not approved.	Not yet approved for clinical use	Refractory asthma characterized by neurogenic inflammation or with comorbid atopic dermatitis (AD).	Possibility of increased skin infection risk necessitates long-term safety evaluation.	(65)
*Anti-IL-9 monoclonal antibodies (Enokizumab )	Targets IL-9 to reduce Tc9/Th2/ILC2-linked mucus and mast-cell support; acts at a peripheral node of the type-2 network with lower endotype prevalence.	Currently in Phase II clinical trials, not approved.	Although in early development, currently lacks robust clinical application data	Potential therapeutic option for patients with refractory asthma accompanied by airway remodeling, particularly those with eosinophil- or mast cell-activated phenotypes	Associated risks include site injection reactions and a potentially increased risk of mild infections	(66, 67)
*JAK/TYK2 inhibitors:	Inhibit JAK1/2/3 and/or TYK2–STAT signaling used by multiple cytokines that shape T-cell programs: dampen Th2/Tfh circuits (IL-4/IL-13 via JAK1/TYK2→STAT6; IL-21 via JAK1/JAK3→STAT3), blunt eosinophil/Th2 effector tone (IL-5 via JAK2→STAT5), and attenuate Th17 maintenance (IL-23 via TYK2/JAK2→STAT3); may also reduce ILC2 amplification via TSLP/IL-9 pathways (JAK1/2; JAK1/3).	Currently in Phase II clinical trials, not approved.	Lack of real-world efficacy data specific to asthma	Th2-high phenotype or mixed phenotype asthma	Infection risk (e. g., herpes zoster) and dyslipidemia	(68)

The drugs marked with an asterisk (*) before their names in the table indicate that the drugs are in the early clinical trial phases.

### 3.2 Recent advances in asthma therapeutics

#### 3.2.1 Novel biologics

Novel biologics have become central to the management of severe asthma. By selectively targeting key inflammatory pathways, these agents significantly reduce exacerbations and inhibit airway remodeling, thereby mitigating the chronic progression of the disease (63, 69). Among currently approved biologics, anti-IL-5/IL-5R agents (mepolizumab, reslizumab, benralizumab) and the IL-4Rα blocker dupilumab deliver consistent benefit in eosinophilic/T2-high disease. The TSLP inhibitor tezepelumab is currently the first epithelial-axis biologic for severe asthma regardless of phenotype, with real-world signals of reduced emergency visits and hospitalizations (69, 71). In contrast, the combinations discussed below (e.g., TSLP plus JAK inhibitors or IL-4Rα antibodies), bispecific antibodies, and strategies to extend dosing intervals are investigational or early-phase and aim to broaden pathway coverage or improve convenience (72–76). Preliminary studies also explore adjunctive allergen immunotherapy and epigenetic associations that may inform future molecular stratification (77–79). Where not explicitly noted as approved, agents in this subsection should be regarded as experimental or in early clinical development.

#### 3.2.2 Small-molecule drugs

Roughly 10–20% of patients with severe asthma respond suboptimally to current therapies, including biologics (80). While biologics are highly effective for T2-high inflammation, their use is constrained by eligible populations and cost (81). Small-molecule drugs therefore provide a complementary option: they are often oral, less costly, and can achieve broad tissue penetration, making them attractive for non-T2 disease and in resource-limited settings (81). Mechanistically, small molecules can target non-T2 inflammatory pathways (e.g., neutrophilic/Th17-linked processes) or epigenetic/enzymatic regulators (such as histone deacetylase inhibition), thereby opening options for patients who are difficult to treat with current biologics (82). To decide which nodes to drug, discovery now moves beyond pathway lists to human-data–driven target prioritization, pairing omics readouts with causal inference so that candidates are anchored in disease biology (82, 83). Emerging discovery strategies integrate metabolomics and Mendelian randomization (MR)—a genetics-based approach that uses inherited variants as natural instruments to test whether changes in a protein are causally related to disease (82, 83). Using this framework, one study nominated IL1R1(Interleukin 1 Receptor Type 1), ECM1(Extracellular Matrix Protein 1), and PDLIM4 (PDZ and LIM Domain Protein 4) as causal asthma targets, with additional signals implicating ECM1 in neuro-immune pathways (83). Within this framework, a protein emerge with both mechanistic plausibility and translational signal—setting the stage for concrete, pathway-directed examples (83, 84). ATF6, a key node in the unfolded protein response (UPR), supports Th2/Th17 differentiation. The ATF6 inhibitor Ceapin A7 suppressed downstream UPR genes and Th cytokines in human and murine memory CD4^+^ T cells, and in chronic asthma models reduced eosinophilia and neutrophilia, indicating potential for steroid-resistant and T2-low asthma (84). the ATF6 inhibitor Ceapin-A7 remains preclinical/early-phase with supportive murine data showing reduced eosinophilia/neutrophilia and potential relevance to steroid-resistant/T2-low asthma (84). Overall, unless otherwise stated, small-molecule examples discussed here are investigational or in early clinical testing, representing pragmatic oral/inhaled candidates that warrant biomarker-enriched trials to realize clinical value.

The role of small molecules is shifting from traditional bronchodilation toward immune-modulating agents that can be used as alternatives or add-ons to biologics. Moving forward, biomarker-enriched trials, pragmatic choices of oral/inhaled delivery, and attention to access and cost will be essential to realize their clinical value.

## 4 Discussion

This review has framed asthma heterogeneity through T-cell axes—Th2/ILC2, Th17/Treg, Tfh/Breg, and CD8^+^ programs—and then mapped approved and investigational therapies onto those pathways (4, 7, 17–34, 36–43).Read together, Sections 2 and 3 indicate a simple organizing principle: when disease biology is dominated by a single axis (e.g., eosinophilic or allergic type-2), axis-matched biologics deliver the most reliable benefit; when signals are mixed or weak, upstream epithelial cues (e. g., alarmins) or combination/sequence strategies are more plausible (47, 48, 50–59, 62–64). The discussion below focuses on what evidence is still missing and how to generate it efficiently. Three issues recur across studies: (1) endotypes are still inferred from single biomarkers rather than integrated signatures; (2) trials often enroll broad populations, diluting signal; and (3) mechanistic work and clinical testing are loosely coupled, so pathway insights do not systematically shape design, dosing, or switching rules (4, 5). Addressing these gaps should be the near-term priority. These gaps point to a focused and tractable agenda. We therefore outline four research priorities that directly remedy them (4, 5, 7–9). (1) Composite, decision-grade biomarkers for endotype assignment:build and validate integrated panels that reflect the T-cell axes reviewed here (e.g., Th2/Tfh vs Th17/Treg balance, epithelial alarmins, CD8^+^ interferon competence). Panels should be calibrated to specific decisions—initiate axis-matched therapy, add an upstream agent, or switch after suboptimal response—rather than to diagnosis alone (4, 7, 17–34, 36–43); (2) Biomarker-enriched and adaptive trial designs:move beyond one-size-fits-all RCTs (Randomized Controlled Trials) by enriching for *a priori* endotypes and using adaptive platforms that allow response-guided randomization, early futility, and mechanism-based switching rules. Trials should pre-specify how biomarker trajectories (e. g., eosinophils, FeNO, IL-17/Treg ratio) control step-up/step-down decisions (4, 7); (3) Therapeutic strategies for T2-low and steroid-insensitive disease:prioritize programs that rebalance Th17/Treg and restore CD8^+^ interferon function, including metabolic rescue approaches, while testing upstream epithelial blockade as a backbone in mixed-signal populations. Where preclinical signals exist (e. g., agents modulating Th17/Treg or neuro-immune inputs), human proof-of-mechanism should use small, biomarker-rich Phase 2 studies before scale-up (27–34, 41–44, 66, 67); (4) Delivery and durability: matching drug format to pathway kinetics:for pathways driven by episodic epithelial stress, long-acting or inhaled formats may better align exposure with site of action; for systemic immune bias, durable systemic agents may be preferable. Comparative studies should test whether route and persistence (SC/IV vs inhaled/oral; long-acting depots) influence real-world control, adherence, and cost (48, 54, 62–64).

The comparative framework in Section 3 and **Table 1** already implies a practical sequence: identify the dominant axis, trial the best-matched agent, and pre-define switch/combination rules when biomarkers and clinical response diverge (47–59). Where late-phase results have been inconsistent (e. g., IL-33/ST2 or IL-9 programs), the likely explanation is endotype dilution rather than absence of biology—reinforcing the need for enrichment and target-engagement checkpoints (44, 56–67). Advanced tools (single-cell/spatial omics, Mendelian randomization, causal proteomics, AI models) should be introduced only insofar as they solve the above priorities: selecting patients, picking doses/targets, and predicting switching benefit (82, 83). Heterogeneity in assays, definitions, and endpoints still hampers between-trial comparisons; many promising approaches remain preclinical or early-phase. We therefore emphasize principles and trial logistics rather than exhaustive target lists (4, 5). Asthma care improves fastest when mechanism-based endotyping is coupled to explicit clinical decision rules. By grounding trials and clinical algorithms in the T-cell axes reviewed here—and by prioritizing composite biomarkers, adaptive designs, T2-low–oriented strategies, and delivery matched to pathway kinetics—the field can translate immunology into predictable selections, switches, and combinations that matter to patients (4, 7–9).
